# Clinical significance of phenotyping and karyotyping of circulating tumor cells in patients with advanced gastric cancer

**DOI:** 10.18632/oncotarget.2175

**Published:** 2014-07-07

**Authors:** Yilin Li, Xiaotian Zhang, Sai Ge, Jing Gao, Jifang Gong, Ming Lu, Qiyue Zhang, Yanshuo Cao, Daisy Dandan Wang, Peter Ping Lin, Lin Shen

**Affiliations:** ^1^ Key Laboratory of Carcinogenesis and Translational Research (Ministry of Education), Department of GI Oncology, Peking University Cancer Hospital & Institute, Beijing, China; ^2^ Cytelligen, San Diego, CA, USA

**Keywords:** gastric cancer, circulating tumor cells, EpCAM-independent enrichment, HER2, aneuploidy

## Abstract

**BACKGROUND:**

Karyotyping and phenotyping of circulating tumor cells (CTCs) in therapeutic cancer patients is of particular clinical significance in terms of both identifying chemo-resistant CTC subtypes and understanding CTC evolution.

**METHODS:**

The integrated subtraction enrichment (SET) and immunostaining-fluorescence *in situ* hybridization (iFISH) platform was applied to detect and characterize CTCs in patients with advanced gastric cancer (AGC). Status of human epidermal growth factor receptor 2 (HER2) expressing and aneuploidy of chromosome 8 in CTCs enriched from the patients was examined by SET-iFISH following clinical chemotherapy or HER2-targeted therapy. CellSearch system was applied as a reference control.

**RESULTS:**

Phenotyping of CTCs in HER2 positive AGC patients demonstrated that HER2^+^ CTCs could be effectively eliminated in response to HER2-targeted therapy. Karyotyping of CTCs indicated that distinct CTCs with different ploidies of chromosomes 8 in AGC patients correlated to either sensitivity or resistance of paclitaxel or cisplatin-based chemotherapy. Examination of the copy number of chromosome 8 in CTCs provides a potential approach for predicting chemotherapeutic efficacy and monitoring chemo-resistance.

**CONCLUSIONS:**

Phenotyping and karyotyping of the enriched CTCs upon ploidy of chromosome 8 or HER2 expression is of clinical potential for monitoring chemo-resistance and evaluating therapeutic efficacy for AGC patients.

## INTRODUCTION

Gastric cancer (GC) is the third leading cause of cancer-related mortality worldwide and is particularly prevalent in Eastern Asia, Eastern Europe, and South America [1, 2]. Recent estimated number of GC-related death toll was 723,000 worldwide in 2012 [1]. Insensitivity to chemotherapy and early development of chemo-resistance of GC lead to both poor survival and limited therapeutic options [3, 4]. In general, less than 60% of GC patients have an effective response to chemotherapy, and the overall 5-year survival rates of GC patients remained as low as only 20% [3, 4]. Identification of a biomarker which may help predict chemotherapy efficacy is therefore of particular significance in terms of improving clinical treatment outcome of GC patients.

Circulating tumor cells (CTCs) are tumor cells shed from primary or metastatic solid tumors into the blood circulation. The American Society of Clinical Oncology (ASCO) has accepted quantification of CTC as a novel breast cancer biomarker [5]. In addition to their demonstrated significant implications on prognosis, CTC is also a promising non-invasive real-time surrogate marker for evaluating therapeutic responses as well as monitoring therapy resistance in patients of various types of tumor [6-11]. It has been reported that enumeration of CTC performed by CellSearch system (Veridex, Raritan, NJ) is applicable for predicting chemotherapy efficacy in metastatic cancers, including breast, prostate, colorectal, and small-cell lung cancer [6-11]. Moreover, a previous prospective study performed on advanced GC (AGC) patients indicated that the post-chemotherapy patients with detectable CTCs had decreased median progression-free survival and overall survival, suggesting that CTC might be a significant surrogate biomarker for predicting chemotherapy efficacy in AGC patients [12].

The principle of CellSearch system to detect CTC is based on the expression of epithelial cell adhesion molecule (EpCAM) on the tumor cell surface and cytokeratins (CKs) in the same tumor cell. However, it has been published elsewhere that CTCs may lose both EpCAM and CKs during epithelial-mesenchymal transition (EMT) [7, 13-15], thus leading to failure of efficient detection of CTCs by EpCAM and CK-dependent strategy. Such inherent bio-characteristics of CTCs greatly restrict clinical application of current strategy to detect CTCs shed from many types of solid tumors. Moreover, further molecular characterization, such as *in situ* genotyping of CTCs, is necessary for identifying the CTC involved in chemo-resistance [6]. However, such characterization could be significantly limited due to perturbing of intracellular signaling pathways (such as Wnt pathway) following cross-linking of EpCAM by anti-EpCAM antibody [16].

In the current study, a strategy of EpCAM-independent subtraction enrichment (SET) [17, 18] integrating with immunostaining-fluorescence *in situ* hybridization (iFISH) was applied to enrich and characterize CTCs in AGC patients. Because overexpression and amplification of human epidermal growth factor receptor 2 (HER2) are eligible for HER2-targeted treatment [19], status of HER2 expression on CTCs in AGC patients was monitored by SET-immunofluorescence staining (SET-IF) and CellSearch in regard to evaluating both eligibility and efficacy for HER2-targeted therapy. In addition, correlation of chemo-resistance to the specific subtypes of CTCs classified upon ploidy of chromosome in AGC patients was investigated.

## RESULTS

### Comparison of CTC enumeration performed by SET-iFISH and CellSearch

**Figure 1:**
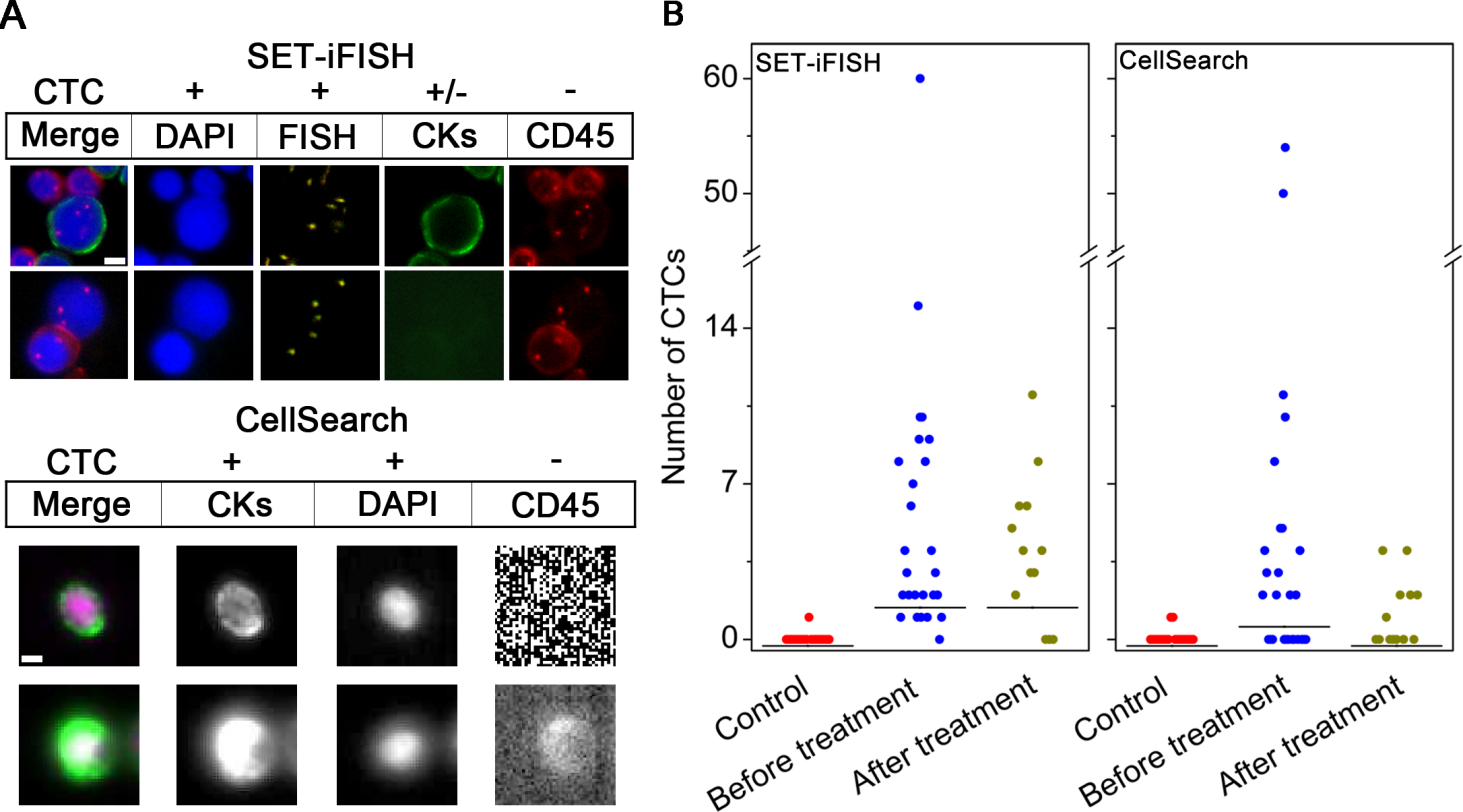
Comparison of CTC enumeration performed by SET-iFISH and CellSearch (A) Images of CTCs revealed by SET-iFISH and CellSearch. CTCs identified by SET-iFISH are DAPI^+^ (blue)/FISH^+^ (aneuploid chromosome 8, orange)/CKs^+ or –^ (green) and CD45^−^; CTC identified by CellSearch are CKs^+^/DAPI^+^/CD45^−^. Scale bar = 5 μm. (B) Enumeration of CTCs by SET-iFISH and CellSearch in healthy donors (Control), AGC patients before and after treatment.

CTCs identified by SET-iFISH and CellSearch are shown in Figure 1A. CTCs demonstrated by SET-iFISH are pan-cytokeratins (PanCK)^+ or −^/4′,6-diamidino-2-phenylindole (DAPI)^+^/FISH^+^ (aneuploid chromosome 8)/CD45^−^; and those identified by CellSearch are CK8,18,19^+^/DAPI^+^/CD45^−^. CTCs with degraded or absent CKs induced by EMT [20] were inevitably ignored by CellSearch, which, however, could be efficiently detected by SET-iFISH.

Enumeration of CTC was performed on 29 AGC patients and 20 healthy donors. Two of 7.5 ml blood were collected from the same patient, followed by subjecting to SET-iFISH and CellSearch, respectively. Shown in Figure 1B, for those patients prior to treatment, the number of CTC detected by SET-iFISH was 0-60 CTCs/7.5 ml, (median number = 4), and 0-54 CTCs/7.5 ml (median number = 2) by CellSearch. Healthy donors showed 0-1 cell/7.5 ml (median number = 0). Difference of CTC number between patients and healthy donors for both systems was significant (both *P* < 0.001). Further analysis on 13 patients who were able to finish 2 cycles of chemotherapy showed that CTC number decreased from 0-60 (median number = 4) to 0-11 cells/7.5 ml (median number = 4) revealed by SET-iFISH, and from 0-54 (median number = 2) to 0-4 cells/7.5 ml (median number = 0) demonstrated by CellSearch. The difference between SET-iFISH and CellSearch was significant (*P* = 0.003), suggesting relatively higher sensitivity of enumeration of CTC performed by SET-iFISH in the post-therapeutic patients.

Concordance of enumeration of CTC between the 2 systems was shown in Table 1. Among the 42 tested samples including 29 prior to and 13 post to treatment patients, CTC positive rate was 90.5% revealed by SET-iFISH and 54.8% shown by CellSearch. The agreement, including both positive and negative CTC counting between the 2 systems was 54.8%, exhibited poor concordance (kappa = 0.020, *P* = 0.001).

**Table 1:** Concordance between SET-iFISH and CellSearch for CTCs enumeration (N = 42)

SET-iFISH	CellSearch		Total (%)
	Positive (%)^a^	Negative (%)	
Positive (%)^a^	21 (50.0)	17 (40.5)	38 (90.5)
Negative (%)	2 (4.8)	2 (4.8)	4 (9.5)
Total (%)	23 (54.8)	19 (45.2)	42 (100.0)
Kappa test	Poor agreement: Kappa = 0.020		
Concordance (%)	23 (54.8), *P* = 0.001		

a Positive CTC is defined as ≥ 1 CTC/7.5 ml

### Correlation of HER2 expression status on CTCs to clinical outcome

**Figure 2:**
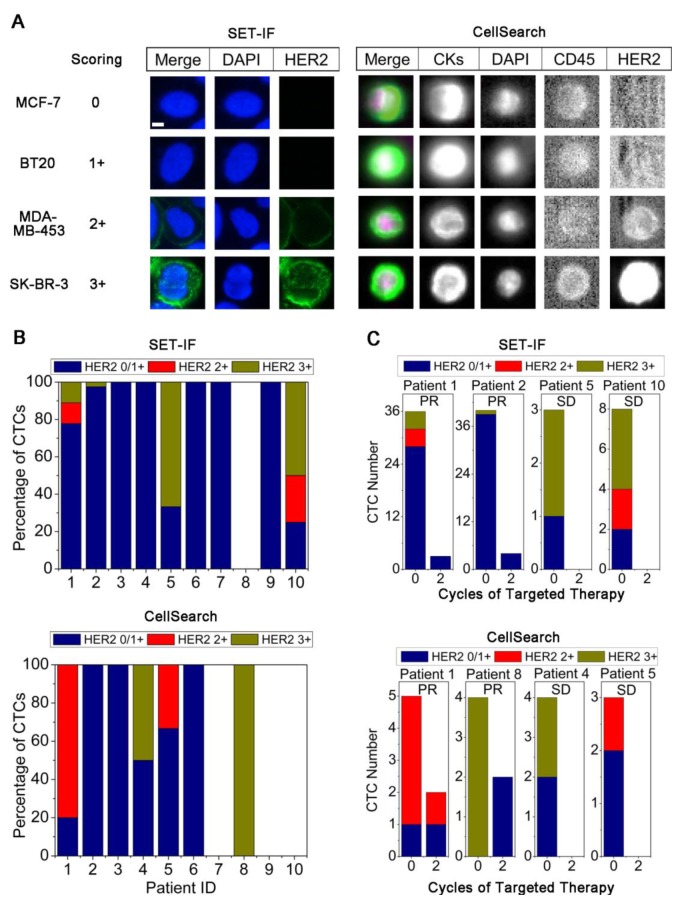
Detection of HER2 expression status on CTCs (A) Quantification of immnuofluorescence stained HER2 on cell lines performed by SET-IF and CellSearch. HER2 expression was quantified by Alexa 488-conjugated anti-HER2 (green) for SET-IF and FITC labeled anti-HER2 for CellSearch. Scale bar = 5 μm. (B) Detection of HER2 expression on CTCs enriched from pathological HER2^+^ AGC patients by SET-IF or CellSearch. (C) Changes of total CTCs and HER2^+^ CTCs following HER2-targeted therapy.

Status of HER2 expression on CTCs was further examined by both SET-IF and CellSearch. To quantify HER2 expression on CTCs, a panel of cell lines with different expression levels of HER2 was examined by both SET-IF and CellSearch. Immunofluorescence intensity of HER2 corresponding to the known HER2 expression status serves as the reference standard for quantification of HER2 status on CTCs [21]. As shown in Figure 2A, immunofluorescence intensity of HER2 was scored as 0 for MCF-7 (none expression), 1+ for BT20 (low), 2+ for MDA-MB-453 (medium), and 3+ for SK-BR-3 (high) cells, respectively. In the case of CTCs, 0 and 1+ were defined as HER2^−^, 2+ and 3+ were classified as HER2^+^.

Primary tumors in 10 out of 29 AGC patients were pathologically identified as HER2^+^. HER2 status on CTCs in those 10 patients was examined by both SET-IF and CellSearch. Shown in Figure 2B, among the 10 HER2^+^ patients, HER2^+^ CTCs were detected in patient 1, 2, 5, and 10 by SET-IF, and in patient 1, 4, 5, and 8 by CellSearch. Both systems had a HER2 CTC positivity rate of 40% (4 out of 10). Two patients (Patient 1 and 5) had HER2^+^ CTCs detected by both platforms. Results of Figure 2B and Supplemental Table 1 indicated that expression of HER2 on different CTCs identified by SET-IF in the same patient or among different patients was highly heterogeneous, scored from 0/1+ to 3+. Similar observation was obtained from CellSearch examination.

All 10 patients with pathological HER2^+^ including those 6 patients showing HER2^+^ CTCs (patient 1, 2, 4, 5, 8 and 10) identified by either SET-IF or CellSearch were subjected to HER2-targeted therapy, and all the patients attained the therapeutic response. Changes of HER2 expression status on CTCs in these 6 patients following HER2-targeted therapy – trastuzumab (TAR) was monitored by SET-IF or CellSearch. As shown in Figure 2C, patient 1, 2, 5, and 10 with HER2^+^ CTCs identified by SET-IF exhibited a dramatic decreased number of CTCs and all of HER2^+^ CTCs were eliminated following 2 cycles of targeted therapy. Similar observation was obtained by CellSearch, showing that, all of HER2^+^ CTCs in patient 4, 5, and 8 were eliminated, and partial elimination from 80.0% down to 50.0% was observed on the patient 1. These results suggest that elimination of HER2^+^ CTCs examined by either SET-IF or CellSearch correlate to the efficacy of HER2-targeted therapy.

### Correlation of aneuploidy of chromosome 8 in CTCs and clinical outcome

**Figure 3:**
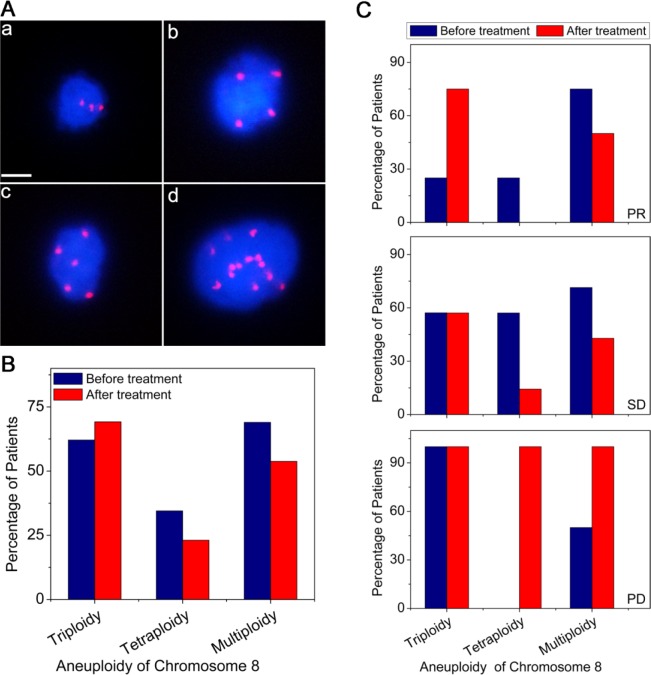
Comparison of ploidy of chromosome 8 in CTCs before and after treatment (A) Images of CTCs with different ploidies of chromosome 8. (a) triploidy, (b) tetraploidy, (c) pentaploidy, and (d) > 5 copies. Nuclei of CTCs were stained with DAPI (blue) and the chromosome 8 was identified by centromere probe 8 SpectrumOrange probe (red dots). Scale bar = 5 μm. (B) Distributions of aneuploid CTCs in AGC patients before and after treatment. (C) Correlation of aneuploid CTCs to the therapeutic efficacy. Multiploidy of chromosome 8 contains pentaploidy and those > 5 copies.

Benefiting from the *in situ* karyotyping of SET-iFISH, aneuploidy of chromosome 8 in CTCs of 29 prior to and 13 post to chemo-treatment AGC patients was examined. As revealed in Figure 3A, triploidy, tetraploidy, pentaploidy or > 5 copies of chromosome 8 in CTCs were observed in AGC patients, indicating existence of heterogeneous polysomic chromosome 8 in CTCs. Figure 3B demonstrated that ratio of CTCs with different ploidies of chromosome 8 was changed following chemotherapy. For those patients prior to treatment, the frequency of CTCs with different chromosome 8 copy number were 62.1% (triploidy), 34.5% (tetraploidy) and 69.0%, (multiploidy). However, decreased frequency of both tetraploidy (34.5% to 23.1%) and multiploidy (69% to 53.8%) of chromosome 8 was observed in CTCs following chemotherapy. Whereas no decreasing of triploid CTC was observed in those post-therapeutic patients (instead of decreasing, the number even slightly increased from 62.1% to 69.2%). Obtained results suggest that triploid chromosome 8 CTCs were not as sensitive as tetraploid and multiploid CTCs to paclitaxel (PTX) or cisplatin (DDP)-based chemotherapy.

Additional analysis was performed on those 13 post-chemotherapeutic AGC patients who were subdivided into 3 groups based on their responses to treatments: partial response (PR, 30% or more decrease in the sum of diameters of target lesions), progressive disease (PD, 20% or more increase in the sum of diameters of target lesions) and stable disease (SD, neither sufficient shrinkage to qualify for PR nor sufficient increase to qualify for PD). Shown in Figure 3C, PR patients had only 25% of triploid chromosome 8 CTCs prior to treatment comparing to SD and PD patients who had triploid CTCs of 57.1% and 100% respectively, suggesting that ratio of triploid CTCs in patients prior to treatment may reciprocally correlate to chemotherapeutic efficacy. The fewer number of triploid chromosome 8 CTCs implies the better chemo-therapeutic efficacy, and vice versa. Obtained results indicated that karyotypic enumeration of triploid CTC may help predict chemotherapeutic efficacy.

Further karyotypic analysis indicated that the ratio of triploid CTCs in those post treatment patients increased from 25.0% (before treatment) to 75.0% (post treatment) in PR patients, or remained the same in SD and PD patients. However, for the tetraploid CTCs, their ratio decreased from 57.1% (before treatment) to 14.3% (post treatment) in SD patients. Similar observation of decreased multiploid CTCs was observed in both PR and SD patients, from 71.4 - 75.0% (before treatment) down to 50.0% (PR) and 42.9% (SD), respectively. For those PD patients, both tetraploid and multiploid CTC reached 100% following chemo-treatment.

In summary, following PTX or DDP-based treatment, in contrast to the ratio of triploid CTCs which was not found to decrease in all treated patients, the percentage of tetraploid and multiploid CTCs decreased in both SD and PR patients, but increased in PD patients. Obtained results suggest that unlike developed or acquired chemo-resistance for tetraploid and multiploid CTCs, the primary chemo-resistance of triploid CTCs is intrinsic.

## DISCUSSION

Clinical validity of the EpCAM and CK-dependent CTC detection has been demonstrated in some solid tumors [6-11]. However, it has been realized that such strategy has great limitation in terms of detection of CTCs shed from various solid tumors due to heterogeneous expression of EpCAM or absence of both EpCAM and CKs induced by EMT [7, 13]. In the current study, the EpCAM-independent SET-iFISH identifying non-hematopoietic heteroploid CTCs was applied to detect and *in situ* characterize CTCs in AGC patients. Comparing to 54.8% CTC positive rate obtained by EpCAM-dependent strategy, SET-iFISH showed 90.5% positive detection in the same population of AGC patients. The 2 strategies showed poor concordance (54.8%, Kappa = 0.020, *P* = 0.001). Reasons accounting for the poor concordance could be that the unbiased enrichment and identification of highly heterogeneous sub-population of CTCs, regardless of EpCAM and CKs expression, lead SET-iFISH a higher CTC detection efficiency comparing to the conventional strategy restricted to only EpCAM^+^/CK^+^ CTCs, thus resulting in a low concordance rate between the 2 technology platforms.

Investigation of HER2 CTCs in AGC patients has been rarely reported [20]. Our study in this paper indicated that HER2 status in AGC patients is very heterogeneous between the primary tumor and CTCs, or even among the individual CTCs of the same patient. Number of HER2^+^ CTCs in AGC patients was reduced or even eliminated following HER2-targeted treatment (Figure 2C), which keeps in agreement with that reported on HER2^+^ breast cancer patients [21-26]. Mechanisms underlying dynamic change of HER2 status on CTC remains to be illustrated. It is reasonable to speculate that apoptosis of HER2^+^ CTCs induced by overexpression of HER2 [25] or hydrolysis of HER2 on CTCs by metalloproteinase [26] may lead to the variation of HER2 expression status on CTCs. As a matter of fact, recent study performed on the xenograft model bearing resistance to TAR also indicated that the HER2^+^ CTCs remained their sensitivities to TAR even after the primary tumor developed resistance to TAR [27]. It is necessary to further expand the current study in order to understand how HER2^+^ CTC develop drug resistance, which will help precisely evaluate and predict HER2^+^-targeted therapeutic efficacy and clinical outcomes.

SET and CellSearch showed 20% (patient 1 and 5) of consistence for HER2^+^ CTC detection (Figure 2B). One of the possibilities accounting for such performance is that some HER2^+^ CTCs identified by SET-IF are EpCAM-negative. The speculation is supported by the published studies showing existence of significant number of EpCAM-negative CTCs during EMT in breast circulating tumor stem cells which have greater metastasizing potential [14]. Extended studies performed on the large amount of clinical samples to further validate and compare SET-IF and CellSearch for their HER2 CTC detection is underway. Our obtained results suggest that multiplex platforms should be integrated to detect HER2 expression on different CTCs subtypes, and evaluate their effectiveness in predicting efficacy of HER2-targeted treatment. In this aspect, the SET-IF can serve as an alternative platform for integrated characterization of HER2 status on CTCs.

Correlation of chromosome 8 ploidy in CTCs to chemotherapy resistance of AGC patients was investigated in this study. Obtained results demonstrated that CTCs with triploid chromosome 8 were constantly resistant to PTX or DDP-based chemotherapy, however, tetra- and multiploid CTCs in the patients who had not been subjected to treatment previously were initially sensitive to the chemotherapy, suggesting that CTCs with triploid chromosome 8 may have intrinsic resistance to PTX or DDP-based chemotherapy, whereas those containing tetra- and multiploidy of chromosome 8 may correlate to the acquired drug resistance. Though preliminary karyotypic characterization of CTCs has been reported by others [28-30], studies described in this paper was for the first time to demonstrate correlation of aneuploidy of chromosomes in CTCs to drug resistance. It has been recently reported that aneuploid colorectal cancer cell lines displayed intrinsic drug resistance compared to diploid/near-diploid cells, suggesting that drug resistance is likely to be an intrinsic property of aneuploid cells [31] rather than a process developed for the acquired drug resistance [32]. Our results in this study suggest that different aneuploidy in CTCs may play distinct role in drug resistance. Triploid chromosome 8 CTCs exhibit intrinsic drug resistance, whereas those with tetra- and multiploid chromosome 8 may develop acquired drug resistance.

In summary, phenotyping and karyotyping of CTCs by SET-iFISH in AGC patients showed that dynamic monitoring HER2 expression status on CTCs could help evaluate efficacy of HER2-targeted therapy in real time. Triploid chromosome 8 CTCs were constantly chemo-resistant, whereas tetra- and multiploid CTCs may develop acquired resistance following chemotherapy. Further mechanistic studies are needed to illustrate this different chemo-sensitivity, though obtained results from this proof-of-concept study suggest that characterization of chromosome 8 aneuploidy in CTCs may be of clinical significance in terms of evaluating and predicting chemotherapeutic efficacy as well as monitoring drug resistance. Extended studies of *in situ* phenotypic and karyotypic characterization of CTC performed by HER2-iFISH on HER2^+^ AGC patients is underway, which will help further illustrate how expressed HER2 and ploidy of chromosome 8 correlate to HER2-targeted therapy.

## MATERIALS AND METHODS

### Patients and sample collection

Twenty nine patients with newly diagnosed AGC, and 20 healthy donors were enrolled at Peking University Cancer Hospital from October 2013 to February 2014. Patients enrolled were histologically confirmed locally advanced or recurrent, and/or metastatic adenocarcinoma of the stomach or gastroesophageal junction. Patients with no prior treatment for advanced/metastatic disease and measurable or nonmeasurable but evaluable disease were included in the study. Patient information was described in Supplemental Table 2. AGC Patients were subjected to the first-line PTX or DDP-based chemotherapy. Drug administration was performed according to the procedure previously published by us [33]. After 2 cycles of chemotherapy, clinical response evaluation was performed with computed tomography (CT) scan according to the RECIST 1.1 criteria. Response assessment was categorized as partial response (PR, at least a 30% decrease in the sum of diameters of target lesions), progressive disease (PD, at least a 20% increase in the sum of diameters of target lesions) and stable disease (SD, neither sufﬁcient shrinkage to qualify for PR nor sufﬁcient increase to qualify for PD). Peripheral blood samples of the enrolled patients and healthy donors were collected prior to and post to 2 cycles of chemotherapy, respectively.

Consent forms in writing to notify blood samples to be applied for the future research were provided to all subjects. The study was approved by the Ethics Committee of Peking University Cancer Hospital and was performed according to the Declaration of Helsinki Principles.

### Detection of CTCs by CellSearch system

Detection of CTCs by CellSearch system was performed as previously described [12]. Briefly, blood samples were drawn into 10-ml CellSave Vacutainer tubes (Becton Dickinson) containing EDTA and cell fixative. Samples were kept at room temperature for up to 72 hours, followed by processing with semi-automated system (CellPrep) and CellSearch Epithelial Cell Kit. Cells expressing EpCAM were captured with antibody-coated ferrous particles, and cell nucleus were labeled with DAPI. Fluorescent dye labeled monoclonal antibodies against leukocytes (CD45-allophycocyan) or cytokeratins (CK 8, 18, 19-phycoerythrin) are applied to distinguish epithelial cells from leukocytes. Further identification and enumeration of CTCs were performed using the CellSpotter Analyzer, a semi-automated fluorescence-based microscopy system that permits computer-generated reconstruction of cellular images. CTCs were defined as nucleated cells with CKs positive and CD45 negative staining. Further characterization of HER2 expression on CTCs by CellSearch system was performed with FITC-labeled anti-HER2 antibody according to the manufacturer instruction (CellSearch tumor phenotyping reagent HER2/neu; Veridex).

### Detection of CTCs by SET-iFISH

Experiment was performed similarly to that previously published and according to the kit instruction (Cytelligen, San Diego, CA, USA) [18]. Briefly, patient blood samples were collected into 7.5-ml tubes containing ACD anti-coagulant (Becton Dickinson, Franklin Lakes, NJ, USA), followed by thorough mixing and overlaying on 3 ml of hCTC separation matrix. Solution was centrifuged at 450 × g for 5 min at room temperature. Supernatants above red blood cells were collected and incubated with immunomagnetic particles conjugated to anti-leukocytes monoclonal antibodies including anti-CD45, CD45 isoforms CD45RA, RB, RC and RO at room temperature for 10 min with gentle shaking. Solution was subsequently subjected to magnetic separation using a magnetic stand (Promega, Madison, WI) to remove leukocytes. The magnetic particle-free solution was spun down at 500 × g for 2 min at room temperature. Sedimented cells were thoroughly mixed with cell fixative and applied onto the coated CTC slides for subsequent iFISH analysis. For CK-iFISH, samples were immunostained with a cocktail of Alexa Fluor 594-conjugated monoclonal anti-CD45 and Alexa Fluor 488-conjugated anti-PanCK (CK4, 5, 6, 8, 10, 13 and 18) or anti-HER2 for 1 h in the dark, followed by FISH performed with Centromere Probe (CEP) 8 SpectrumOrange (Vysis, Abbott Laboratories, Abbott Park, IL, USA) using a S500 StatSpin ThermoBrite Slide Hybridization/Denaturation System (Abbott Molecular, Des Plaines, IL, USA). CTCs were identified as DAPI^+^/CD45^−^/PanCK^+^^or^^−^ or HER2^+^ with aneuploid chromosome 8.

### Detection of HER2 expression on cell lines

Breast cancer cell lines including MCF-7, BT20, MDA-MB-453 and SK-BR-3, each with different amount of HER2 expression, were obtained from Cell Resource Center, Chinese Academy of Medical Science & Peking Union Medical College (Beijing, China), and cultured as previously described [21]. To detect HER2 expression by CellSearch system, approximate 500 cells were added into the blood of healthy donors and subsequently processed under identical conditions using the CellSearch system. For quantification of HER2 by SET-IF, approximate 1 × 10^4^ cells of each cell line were applied onto the coated CTC slides to have a monolayer, followed by immunostaning and analysis of HER2 performed on the individual cell as described above.

### Statistical analysis

All statistical analyses were performed with SPSS 18.0 software. The differences in CTC number between patients and healthy donors or between SET-iFISH and CellSearch systems were compared by Mann-Whitney U test. Correlation between the two systems was assessed by Kappa and McNemar's tests. *P* < 0.05 was statistically significant. All the *P* values were two-sided.

## SUPPLEMENTARY MATERIAL TABLES


