# Supplementary material for: Clinical significance of phenotyping and karyotyping of circulating tumor cells in patients with advanced gastric cancer
Source: Oncotarget. 2014 Jul 7;5(16):6594–602. doi: 10.18632/oncotarget.2175 (PMC4196148; doi:10.18632/oncotarget.2175)
Supplement: Supplementary file 1 [file oncotarget-05-6594-s001.doc]

Clinical significance of phenotyping and karyotyping of circulating tumor cells in patients with advanced gastric cancer

**Supplemental Table 1: Distribution of CTCs with different HER2 immunostaining score analyzed by SET-IF and CellSearch.**

| HER2 staining score of CTCs | Number of patients by SET-IF | Number of patients by CellSearch |
| --- | --- | --- |
| CTC (0/1+) | 5 | 3 |
| CTC (2+) | 0 | 0 |
| CTC (3+) | 0 | 1 |
| CTC (0/1+ and 2+) | 0 | 2 |
| CTC (0/1+ and 3+) | 2 | 1 |
| CTC (2+ and 3+) | 0 | 0 |
| CTC (0/1+, 2+ and 3+) | 2 | 0 |
| HER2 positive^a^ | 4 | 4 |
| Total^b^ | 9 | 7 |

^a^HER2 positive is defined as ≥ 1 CTCs/7.5 ml with 2^+^ or 3^+^ HER2 expression.

^b^Total: samples with ≥ 1 CTCs/7.5 ml.

**Supplemental Table 2:** Patient characteristics

| Characteristics | Evaluable Patients (N = 29) | |
| --- | --- | --- |
|  | No. | % |
| **Age at baseline, years** |  | |
| Median | 63 | |
| Range | 49-78 | |
| **Sex** |  |  |
| Female | 10 | 34.5 |
| Male | 19 | 65.5 |
| **Primary tumor site** |  |  |
| Stomach | 16 | 55.2 |
| Gastroesophageal junction | 13 | 44.8 |
| **Lauren classification** |  |  |
| Intestinal | 17 | 58.6 |
| Diffuse | 5 | 17.2 |
| Mixed | 6 | 20.7 |
| **Sites of metastasis** |  |  |
| Liver | 10 | 34.5 |
| Lung | 8 | 27.6 |
| Bone | 1 | 3.4 |
| Peritoneum | 4 | 13.8 |
| Lymph node | 25 | 86.2 |
| **HER2 status** |  |  |
| Positive | 10 | 34.5 |
| Negtive | 16 | 55.2 |
| **Type of treatment^a^** |  |  |
| No treatment | 2 | 6.9 |
| PTX-based | 12 | 41.4 |
| DDP-based | 13 | 44.8 |
| Others | 2 | 6.9 |

^a^PTX-based: PTX alone; PTX plus capecitabine (CAPE). DDP-based: oxaliplatin (OXA) or DDP plus S-1 or CAPE; OXA or DDP plus CAPE and trastuzumab (TRA); Others: CAPE alone or TRA alone.
